# Analysis of magnetic resonance contrast agent entrapment following reversible electroporation *in vitro*

**DOI:** 10.2478/raon-2024-0047

**Published:** 2024-09-15

**Authors:** Marko Strucic, Damijan Miklavcic, Zala Vidic, Maria Scuderi, Igor Sersa, Matej Kranjc

**Affiliations:** Faculty of Electrical Engineering, University of Ljubljana, Ljubljana, Slovenia; Jožef Stefan Institute, Ljubljana, Slovenia

**Keywords:** electroporation, membrane permeabilization, magnetic resonance contrast agent, T_1_ relaxometry

## Abstract

**Background:**

Administering gadolinium-based contrast agent before electroporation allows the contrast agent to enter the cells and enables MRI assessment of reversibly electroporated regions. The aim of this study was evaluation of contrast agent entrapment in Chinese hamster ovary (CHO) cells and comparison of these results with those determined by standard *in vitro* methods for assessing cell membrane permeability, cell membrane integrity and cell survival following electroporation.

**Materials and methods:**

Cell membrane permeabilization and cell membrane integrity experiments were performed using YO-PRO-1 dye and propidium iodide, respectively. Cell survival experiments were performed by assessing metabolic activity of cells using MTS assay. The entrapment of gadolinium-based contrast agent gadobutrol inside the cells was evaluated using T_1_ relaxometry of cell suspensions 25 min and 24 h after electroporation and confirmed by inductively coupled plasma mass spectrometry.

**Results:**

Contrast agent was detected 25 min and 24 h after the delivery of electric pulses in cells that were reversibly electroporated. In addition, contrast agent was present in irreversibly electroporated cells 25 min after the delivery of electric pulses but was no longer detected in irreversibly electroporated cells after 24 h. Inductively coupled plasma mass spectrometry showed a proportional decrease in gadolinium content per cell with shortening of T_1_ relaxation time (*R*^2^ = 0.88 and *p* = 0.0191).

**Conclusions:**

Our results demonstrate that the contrast agent is entrapped in cells exposed to reversible electroporation but exits from cells exposed to irreversible electroporation within 24 h, thus confirming the hypothesis on which detection experiments *in vivo* were based.

## Introduction

Exposure of cells to short high-voltage electric pulses, if sufficiently high, can cause an increase of cell membrane permeability. This phenomenon, known as electroporation, allows transport of otherwise impermeable molecules (including hydrophilic molecules, such as chemotherapeutic drugs, and large molecules, such as RNA, DNA, etc.) across the membrane. If the cell membrane reseals after exposure to electric pulses, molecules remain entrapped inside the cell. This phenomenon is termed reversible electroporation, if cells preserve their viability.^1^ Cell membrane electroporation can also result in cell death, which is known as irreversible electroporation.^2,3^ In medicine, electroporation-based treatments and therapies utilize reversible electroporation in electrochemotherapy and gene electrotransfection treatments, while irreversible electroporation is used as tissue ablation treatment.^4,5,6^

Electroporation can be considered a threshold phenomenon, i.e. if a specific cell is exposed to an electric field above certain value using set pulse parameters, it will determine both whether electroporation occurs and reversibility of this phenomenon.^7,8,9,10^ Thresholds are simplified concepts assuming electroporation to be a discrete phenomenon. However, cell membrane permeability changes due to exposure to electric field are continuous and depend on the strength of electric field and exposure time.^11,12^ It has also been shown that for different cell types^13,14^ tissue type^11,15^ and different pulse protocols^16,17,18^ different electric field strengths values are needed, i.e. different threshold apply. Successful outcome of both reversible electroporation^19^ and irreversible electroporation^20^ is thus not easy to predict.

Electroporation *in vitro* can be determined using various methods, including voltage clamp techniques^21^, microscopy^22^ and most commonly, by detecting a reporter molecule due to increase of molecular transport across the membrane.^23^ Latter detection methods are often based on exogenous reporter molecules (propidium iodide, trypan blue, lucifer yellow) and on functional molecules that can be detected inside the cell (DNA, RNA) or cause cell death (cisplatin, bleomycin).^23^ In contrast, determining electroporation *in vivo* has proven to be more challenging, with fewer available methods. Electric field distribution is difficult to predict *in vivo*^24,25,26^ and electroporation treatment outcome becomes evident weeks after the treatment.^27,28,29^ One of potentially interesting approaches proposed is using hydrophilic gadolinium-based contrast agent (CA) to visualize reversible electroporation *in vivo* using MRI.^30,31^ When CA is administered prior to electroporation, CA can enter the cell during electroporation and become entrapped once the cell membrane reseals, i.e. in reversibly electroporated cells. After CA is washed from the body a decrease of T_1_ relaxation times in areas where CA is entrapped can be visualized using MRI.^30,31^ This approach was successfully used on follow up studies to assess reversibly electroporated regions *in vivo*^7,31,32^, however, the hypothesis on which this approach is based have not yet been evaluated *in vitro*. Therefore, in our study, we focused on the *in vitro* evaluation of CA entrapment in cells exposed to different amplitudes of electric pulses to achieve either reversible or irreversible electroporation. We compared these results with those obtained using standard *in vitro* methods: YO-PRO-1 dye for assessing cell membrane permeability due to electroporation, propidium iodide fluorescent dye for cell membrane integrity, and the MTS assay for cell survival assessment.

## Materials and methods

**FIGURE 1.**
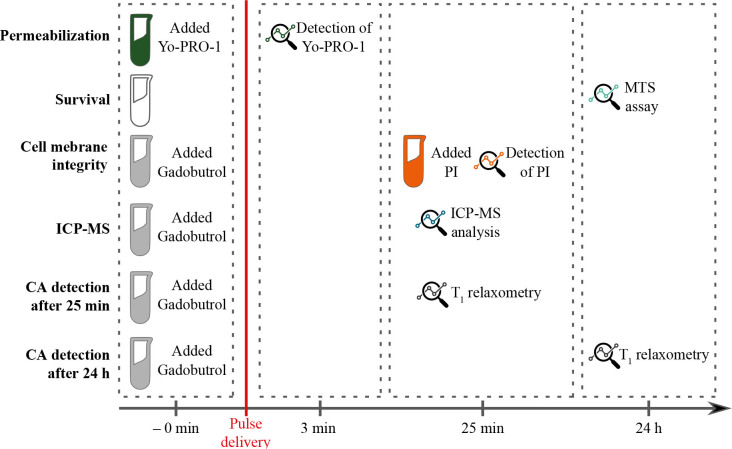
An overview of the time sequence of experiments. Red line represents a moment of pulse delivery. For cell membrane integrity, inductively coupled plasma mass spectrometry (ICP-MS), and Gadolinium-based contrast agent (CA) detection experiments gadobutrol was added to cell suspension prior to pulse delivery. Analyses were performed at different time points as indicated in the figure. PI = Propidium iodide

An overview of the time sequence of different experiments performed in the study is shown in Figure 1. Permeabilization experiments were performed using YO-PRO-1 dye which was added before the delivery of electric pulses and the presence of YO-PRO-1 inside the cells was determined immediately after pulse delivery. Cell survival was determined 24 h after pulse delivery by MTS assay. Gadolinium-based contrast agent (CA) gadobutrol was added before delivery of electric pulses for the rest of the experiments. Cell membrane integrity was assessed 25 min after pulse delivery with propidium iodide. At the same time point, the presence of CA inside of the cells was evaluated using inductively coupled plasma mass spectrometry (ICP-MS). CA detection in cell suspensions using T_1_ relaxometry was performed 25 min and 24 h after pulse delivery.

### Cell preparation

Chinese hamster ovary (CHO-K1) cell line was obtained from the European Collection of Authenticated Cell Cultures (ECACC, cat. no. 85051005). Cells were grown in F-12 Ham nutrient mixture (cat. no. N6658, Sigma-Aldrich, MO, United States) supplemented with 10% fetal bovine serum (FBS, cat. no. F9665, Sigma-Aldrich), 1 U/ml penicillin/streptomycin (cat. no. P0781, Sigma-Aldrich) and 50 μg/ml gentamycin (cat. no. G1397, Sigma-Aldrich) (i.e. complete growth medium) at 37°C in a humidified, 5% CO_2_ atmosphere. For the experiment, cells were detached with trypsin solution 10 × trypsin-EDTA (PAA, Leonding, Austria) and 1:9 diluted in Hank’s basal salt solution (StemCell, BC, Canada). After cells were detached, trypsin was inactivated by complete growth medium. Cells were transferred to a 50 ml centrifuge tube and centrifuged 5 min at 200 g at room temperature. The supernatant was aspirated, and cells were resuspended Dulbecco’s Modified Eagle Medium (DMEM, cat. no. D5671, Sigma-Aldrich) supplemented with 10% fetal bovine serum (FBS, cat. no. F9665, Sigma-Aldrich), 1 U/ml penicillin/streptomycin (cat. no. P0781, Sigma-Aldrich) and 50 μg/ml gentamycin (cat. no. G1397, Sigma-Aldrich) (i.e. electroporation medium) as in Vižintin *et al*., 2021.^17^ Such medium was used for permeability assay and ICP-MS, while for other assays also 10 mM HEPES buffer (cat. no. H3375, Sigma-Aldrich) was added to electroporation medium. Cell volume fraction of 7% corresponding to the final concentration of 8.9×10^7^ cells/ml was used in all experiments.

### Delivery of electric pulses

For delivery of electric pulses 150 μl of cell suspension was transferred to cuvette with parallel aluminum plate electrodes (d = 2 mm, VWR, Radnor, PA, USA). Pulse protocol (8 pulses of 100 μs, delivered at a pulse repetition rate of 1 Hz) was delivered with the prototype pulse generator L-POR V0.1 (mPOR, Ljubljana, Slovenia). Delivery of electroporation pulses was monitored using HDO6000 high-definition oscilloscope (Teledyne LeCroy, Chestnut Ridge, NY, USA), a high-voltage differential probe HVD3605A (Teledyne LeCroy) and current probe CP031 (Teledyne LeCroy). Electric field (E) was calculated as E = U/d where d equals distance between aluminum plate electrodes in cuvettes (2 mm) and U equals delivered voltage. Pulse delivery parameters are presented in Table 1.

**TABLE 1.** Parameters of electric pulses used in experiments

**Experiment**	**U [V]**	**E [kV/cm]**	**Single pulse duration [μs]**	**Pulse repetition rate [1/s]**	**Number of pulses [/]**
Permeabilization	120–400	0.6–2.0	100	1	8
ICP-MS	120–280	0.6–1.4	100	1	8
Cell survival	160–600	0.8–3.0	100	1	8
Cell membrane integrity	160–600	0.8–3.0	100	1	8
CA detection experiments	160–600	0.8–3.0	100	1	8

CA = contrast agent; ICP-MS = inductively coupled plasma mass spectrometry

### Permeabilization experiments

Prior to experiments, YO-PRO-1 (cat. no Y3603, Thermo Fisher Scientfic, Waltham, MA, USA) was added to sample to obtain the concentration of 1μM YO-PRO-1. After pulse delivery, 20 μl of the cell suspensions was transferred to a 1.5 ml centrifuge tube and incubated for 3 min at room temperature. After incubation, cells were diluted with 150 μL of fresh electroporation medium, and YO-PRO-1 uptake was detected with a flow cytometer (Attune NxT, Life Technologies, Carlsbad, CA, USA using blue LED laser (wavelength: 488 nm), and a 530/30 nm band-pass filter. The analysis of 10,000 events was performed by the Attune Nxt software. On the dot-plots of forward-scatter and side-scatter, cell debris and (cell) clusters were excluded from the analysis. Fluorescence intensity histograms were used to determine the percentage of YO-PRO-1 permeabilized cells. Gating was set according to sham control (0 V).

### MTS survival assay experiments

For survival experiments 25 min after pulse delivery, 10 μl of cell suspension was diluted in 4 mL Ham-F12 growth medium. After that, 100 μl of sample was transferred to 96-well plate in triplicates. Plates were incubated at 37°C in a humidified, 5% CO_2_ atmosphere for 24 h. According to manufacturer’s instructions (CellTiter 96 AQueous One Solution Cell Proliferation Assay, Promega, Madison, WI, USA), 20 μL of MTS tetrazolium compound was added to the samples, and after 2 h the absorbance of formazan (reduced MTS tetrazolium compound) was measured with a spectrofluorometer (Tecan Infinite M200, Tecan, Grödig, Austria) at 490 nm. The percentage of viable cells was obtained by the normalization of sample absorbance to the absorbance of the control (0 V).

### Cell membrane integrity experiments

Prior to pulse delivery, cells were mixed with gadolinium-based contrast agent gadobutrol (Gadovist^®^ 1.0 mM, Bayer, Leverkusen, Germany) to a final concentration of 22 mM, then 150 μl of sample was transferred to cuvettes. After pulse delivery, cells were incubated at room temperature for 25 min. After incubation, 20 μl of cell suspension was diluted in 150 μl of fresh growth medium. Propidium iodide (PI, cat. no BMS500PI, Thermo Fisher Scientfic) was then added to the sample to the final concentration of 100 μg/ml and cells were incubated at room temperature for another 5 min. This was followed by analysis of PI uptake on flow cytometer using blue LED laser (wavelength: 488 nm) and a 574/26 nm band-pass filter. The analysis of 10,000 events was performed by the Attune Nxt software. On the dot-plots of forward-scatter and side-scatter, cell debris and (cell) clusters were excluded from the analysis. Fluorescence intensity histograms were used to determine the percentage of PI permeabilized cells. Gating was set according to sham control (0 V).

### Cell suspension preparation for gadolinium-based contrast agent detection experiments

Prior to pulse delivery, cells were mixed with gadobutrol (Gadovist^®^ 1.0 mM, Bayer, Leverkusen, Germany) to a final concentration of 22 mM, then 150 uL of sample was transferred to cuvettes, 125 μl of the cell suspension was transferred to 5 ml of fresh growth medium 25 min after pulse delivery for the washing steps. Cells were centrifuged for 5 min at 900 g to separate the gadobutrol entrapped in the cells from the medium. Then medium was removed, and cells were resuspended in 2 ml of fresh growth medium, and the centrifugation step was repeated. This washing step was repeated two times. At the end cells were resuspended in 900 μl of fresh growth medium, to achieve 1% cell volume fraction for T_1_ relaxometry analysis.

For CA detection experiments at 24 h after pulse delivery, same steps as described above were performed, however, after last centrifugation step cells were seeded in 20 ml of growth medium in T150 cell culture flasks (TPP, Switzerland) for 24 h at 37°C in a humidified 5% CO_2_ atmosphere. Afterwards, growth medium from each culture flask was collected in 50 ml centrifuge tube. Cells were then detached with trypsin solution 10 × trypsin-EDTA (PAA) and 1:9 diluted in Hank’s basal salt solution (StemCell). Trypsin was inactivated by fresh growth medium. Cells were then harvested and added to previously collected growth medium in a 50 ml centrifuge tube. The centrifugation step was then repeated as in the previous day and the cells were again resuspended in 900 μL of fresh growth medium for T_1_ relaxometry analysis.

### Gadolinium-based contrast agent detection experiments

Nuclear Magnetic Resonance (NMR) scanner was used for determining T_1_ relaxation times of cell suspensions. NMR scanner included a 2.35 T horizontal bore superconducting magnet with resonant proton frequency of 100 MHz (Oxford Instruments, Abingdon, UK) connected to a Redstone spectrometer (Tecmag, Houston TX, USA) and equipped with microimaging accessories with maximum gradients of 250 mT/m (Bruker, Ettlinger, Germany). T_1_ relaxometry was performed using inverse recovery spectroscopic pulse sequence in multiple points along the z axis of the sample with variable repetition rates. Relaxation times were then calculated from the signal intensities in OriginPRO 2024 (OriginLab Corporation, Northampton, MA, USA) using 3 parameter exponential fitting curve using fitting function: *M_z_* = *M*_0_ − Δ*Me*^−*TR*/*T*_1_^, where *M*_z_ is measured longitudinal magnetization, *M*_0_ is initial longitudinal magnetization at equilibrium, *ΔM* is the maximum magnetization difference from equilibrium, *TR* is repetition time and *T*_1_ is longitudinal relaxation time.

### Inductively coupled plasma mass spectrometry experiments

For determination of intracellular concentration of gadolinium (Gd), the cell pellet with 1 ×10^7^ cells was separated from the supernatant after electroporation and analyzed using inductively coupled plasma mass spectrometry (ICP-MS). To aid sample digestion, 0.1 ml of H_2_O_2_ and 0.1 ml of HNO_3_ (both from Merck, Darmstadt, Germany), were added to the cell pellets. The tubes were then sealed with caps and Teflon tape and left overnight at 80°C. Following digestion, 1.8 ml of Milli-Q water (Direct-Q 5 Ultrapure water system; Merck Millipore, MA, USA) was added. Gadolinium in samples was then measured using ICP-MS (7900 ICP-MS; Agilent Technologies, California, USA) with Gadolinium ICP standard (cat. no. 170318, Merck) used as an internal standard during the measurement. To determine the amount of Gd per cell, the number of cells in the pellet was divided with the measured Gd in the cell pellet of each sample. Control samples (cells which were not electroporated and were not incubated with gadobutrol) were used for blank subtraction for all gadobutrol-treated samples. To reduce cross-contamination of the instrument during the measurement, a mixture containing 1% HNO_3_ and 1% HCl (Merck) was used as a rinse between the sample runs.

### Statistical analysis

Significant differences were evaluated by the Welch Two Sample t-test at a significance level of 95% (p < 0.05). Statistical analysis was performed using MATLAB 2021b (MathWorks, Natick, MA, USA).

## Results

In our study we tested the hypothesis that contrast agent (CA) is entrapped inside reversibly electroporated cells. Measurement results of CA by T_1_ relaxometry and inductively coupled plasma mass spectrometry (ICP-MS) in cells *in vitro* were compared to results obtained by established methods for assessing cell membrane permeabilization, cell membrane integrity and cell survival. As expected, CA was detected 25 min and 24 h after the delivery of electric pulses in cells that were reversibly electroporated. In addition, CA was present in irreversibly electroporated cells 25 min after the delivery of electric pulses but was no longer detected in irreversibly electroporated cells after 24 h.

### Permeabilization and survival

**FIGURE 2.**
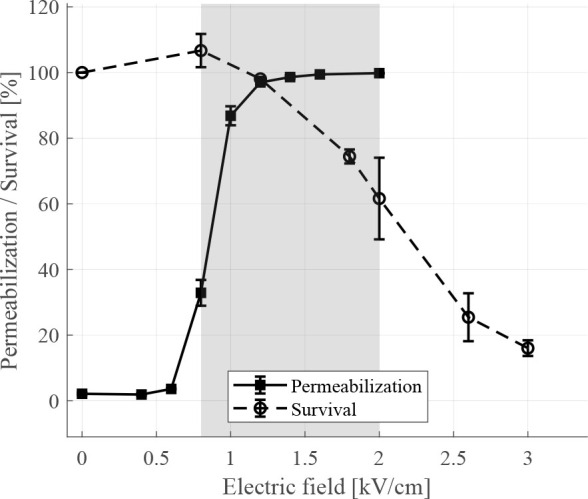
Cell membrane permeabilization (solid black line) and cell survival (dashed black line) of Chinese hamster ovary (CHO) cells in relation to applied electric field. Cell membrane permeabilization and cell survival experiments were performed using YO-PRO-1 dye and by assessing metabolic activity of cells using MTS assay, respectively. Each data point presents a mean ± standard deviation (vertical bars) of 3 repetitions. For permeabilization results gating was set according to sham control without applied electric field. Survival results are normalized to the control sample without applied electric field. Area shaded in gray represents range of electric fields which predominantly cause reversible electroporation of cells.

As shown in Figure 2, results of permeabilization experiments using YO-PRO-1 dye show increase in cell membrane permeability with increased pulse amplitude starting between 0.6 and 0.8 kV/cm at which 32.88 ± 3.93% of CHO cells were permeabilized, while at 1.2 kV/cm nearly all cells (96.99 ± 0.45%) in cell suspension were permeabilized. Cell survival, as determined by MTS assay performed at 24 h after the delivery of electric pulses, shows 61.61 ± 12.44% of cells survived when exposed to the electric field of 2.0 kV/cm. Survival at higher pulse amplitudes further decreased. Using these results, the range of electric fields which predominantly cause reversible electroporation was set between 0.8 kV/cm and 2.0 kV/cm (gray shaded area in Figure 2).

### Cell membrane integrity

**FIGURE 3.**
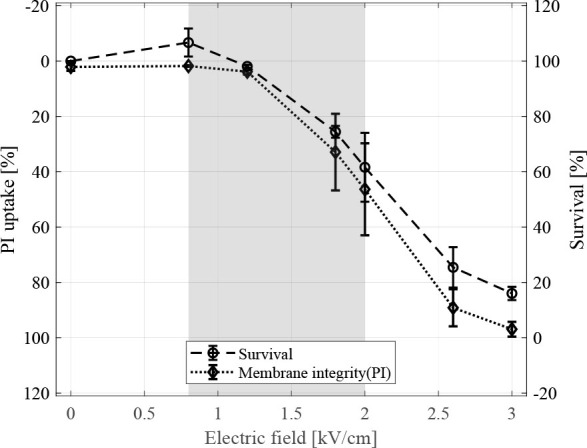
Cell membrane integrity experiment determined by adding propidium iodide dye 25 min after pulse delivery and cell survival determined by MTS assay 24 h after pulse delivery in relation to applied electric field. Each data point presents a mean ± standard deviation (vertical bars) of 3 repetitions. For cell membrane integrity results gating was set according to sham control without applied electric field. Survival results are normalized to the control sample without applied electric field and with added 22 μM of gadobutrol. Note the reversed (upside -down) scale of propidium iodide (PI) uptake for easier comparison. Area shaded in gray represents range of electric fields which predominantly cause reversible electroporation of cells.

Cell membrane integrity was determined by adding propidium iodide to cell suspensions 25 min after pulse delivery and measuring propidium iodide inside CHO cells by flow cytometry (Figure 3, dotted curve). Propidium iodide uptake into the cells after membrane resealing showed that majority of cells can restore membrane integrity at electric fields lower than 0.8 kV/cm up to which only 1.80 ± 0.26% were stained with propidium iodide. While at electric fields above 2.0 kV/cm cell membrane integrity was no longer restored in 46.34 ± 16.62% of cells (Figure 3, dotted curve). For comparison, a cell survival curve obtained by MTS assay at 24 h from Figure 2 is added in Figure 3 (dashed curve).

### T_1_ relaxation times

**FIGURE 4.**
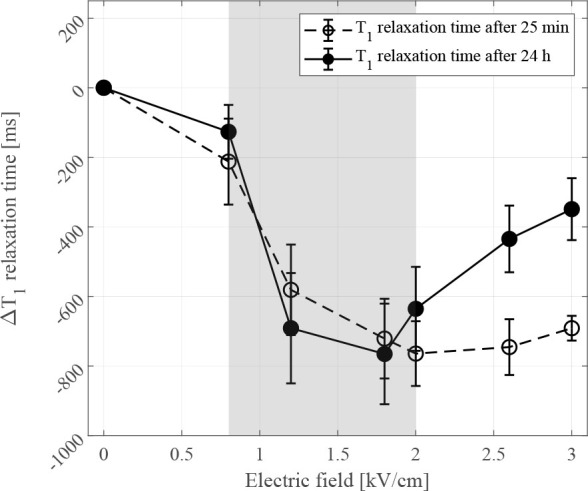
Change in T_1_ relaxation times obtained from CHO cells 25 mins (dashed line) and 24 h (solid line) after pulse delivery. Each data point presents a mean ± standard deviation (vertical bars) of 3 repetitions. Comparison of T_1_ relaxation times obtained 25 mins and 24 h after electroporation (EP) is normalized to control sample, i.e. cell suspension with added 22 μM gadobutrol and without exposure to an electric field. Asterisks (*) indicate statistically significant differences (p < 0.05) between T_1_ relaxation time curves obtained 25 min and 24 h after pulse delivery. Area shaded in gray represents a range of electric fields which predominantly cause reversible electroporation of cells.

T_1_ relaxation times of cell suspensions measured 25 mins after the delivery of electric pulses, began to shorten at 0.8 kV/cm compared to the control and continued to decrease until reaching a plateau at electric field of 1.8 kV/cm (Figure. 4 dashed line). T_1_ relaxation times of cell suspensions, measured 24 h after the delivery of electric pulses (Figure 4 solid line), showed a similar shortening of T_1_ relaxation times as observed when measured 25 min after pulse delivery up to an applied electric field of 1.8 kV/cm. However, from 1.8 kV/cm up to 3 kV/cm, T_1_ relaxation times of cells measured 24 h after the delivery of electric pulses started to increase compared to cells measured at 25 min (Figure 4).

### Inductively coupled plasma mass spectrometry

**FIGURE 5.**
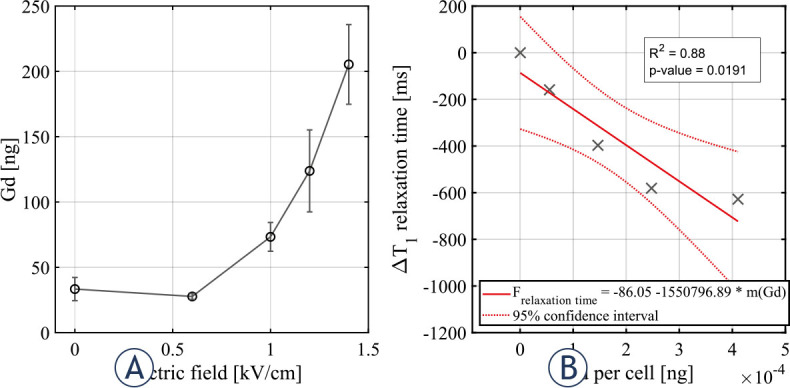
Mass of gadolinium per cell in relation to applied electric field 25 min after pulse delivery. Each data point presents a mean ± standard deviation (vertical bars) of 3 repetitions **(A)**. Linear regression fitting of T1 relaxation time in relation to mass of gadolinium per cell. Each symbol represents a point extrapolated from T1 relaxometry results 25 min after pulse delivery **(B)**.

To confirm presence of CA (gadobutrol) inside CHO cells after electroporation, inductively coupled plasma mass spectrometry (ICP-MS) analysis was performed 25 min after pulse delivery. Results showed Gd (a paramagnetic core of gadobutrol) was present in increased quantities in cells exposed to electric fields ranging from 0.6 kV/cm to 1.4 kV/cm (Figure 5A). Note that electric field of 1.4 kV/cm, 100% permeabilization was achieved, while cell survival remained unaffected (Figure 2). Based on these results, the gadolinium content per cell was determined by dividing measured gadolinium mass by the number of cells (1×10^7^) in the pellet. Change in T_1_ relaxation times were extrapolated from T_1_ relaxometry experiment performed 25 min after pulse delivery. As shown in Figure 5B, linear regression analysis showed a proportional decrease in gadolinium content per cell with shortening of T_1_ relaxation time (R^2^ = 0.88 and p-value = 0.0191).

## Discussion

Gadolinium-based contrast agent gadobutrol (CA) is unable to enter cells under physiological conditions and are rapidly eliminated from the body. The mean elimination half-life of gadobutrol is 1.8 h, which corresponds to the renal elimination rate in healthy individuals. CA are traditionally used in magnetic resonance imaging to increase sensitivity and specificity of diagnostic images enhancing regions with increased perfusion and edema.^33^ However, if CA is present in tissue prior to electroporation it can enter cells after pulse delivery and remain entrapped inside reversibly electroporated cells which has been used as threshold determinant in several *in vivo* studies.^30,31,34^ Entrapped CA can be detected 24 h – 72 h after injection of CA and electroporation *in vivo*, after remaining CA, i.e. extracellular CA has been eliminated from the body.

In this study, we tested the basic assumption of CA entrapment *in vitro* using CHO cells exposed to different amplitudes of electric pulses. To evaluate CA entrapment in relation to reversible and irreversible electroporation, CA detection by T_1_ relaxometry and ICP-MS findings were compared to results obtained from established methods for assessing cell membrane permeabilization, cell membrane integrity and cell survival, i.e. for determining range of reversible electroporation. Thus, determined electric fields for CA uptake detection experiments were ranging from 0.8 kV/cm, where the first significant permeabilization was detected, to 3.0 kV/cm, where cell survival was no longer expected according to cell survival results (Figure 2). The presence of CA in cells was also confirmed by inductively coupled plasma mass spectrometry (ICP-MS) (Figure 5).

Cell membrane permeability determined by the YO-PRO-1 dye reached a plateau within the range of electric fields from 1.0 kV/cm to 1.2 kV (Figure 2). Conversely, results obtained from ICP-MS experiments show increasing amounts of Gd up to an electric field of 1.4 kV/cm (Figure 5A). We therefore extended our investigation by comparing the results of permeabilization and CA detection experiments to higher pulse amplitudes. Comparison showed plateau from T_1_ relaxometry results is shifted towards higher electric fields between 1.2 kV/cm and 1.8 kV/cm (Figure 4) compared to permeabilization results. The observed plateau shift could indicate different kinetics of transmembrane transport for different molecules. But it is also important to consider the methodology used in permeabilization experiments. In permeabilization experiments we determined a fraction of permeabilized cells, i.e. YO-PRO-1 positive cells in suspension, whereas in both ICP-MS and T_1_ relaxation experiments, the presence of total CA in suspension was determined, allowing accumulation of CA in individual cells at electric fields above those needed for permeabilization of all cells, which can have an additional impact on the T_1_ relaxation time shortening.

Interestingly, T_1_ relaxation times at 25 min after pulse delivery remained decreased even at higher electric fields than irreversible threshold, e.g. at 2.6 kV/cm and 3.0 kV/cm (Figure 4), suggesting presence of CA even in cells that are irreversibly electroporated. The MTS survival assay performed 24 h after electroporation showed most cells exposed to electric field between 2.6 kV/cm and 3.0 kV/cm die due to irreversible electroporation (Figure 2). To investigate if the presence of CA in cells exposed to irreversible electroporation is related to transient membrane resealing before eventual cell death, evaluation of cell membrane integrity using propidium iodide was performed 25 min after pulse delivery. The results of cell membrane integrity experiments show good agreement with MTS survival assay (Figure 3) which confirmed lack of cell membrane integrity of cells exposed to irreversible electroporation at 25 min after pulse delivery. Note the cell death can be delayed which is related to varying levels of membrane damage after electroporation.^35^ The results of our study with respect to cell membrane integrity are also in agreements with the reported times of 10–15 min for cell membrane resealing for pulse amplitudes in ranges of reversible electroporation.^36,37,38,39^ Thus, entrapped CA is unable to exit reversibly electroporated cells after 25 min but should be able to exit irreversibly electroporated cells. Since presence of CA in cells exposed to electric fields in range of irreversible electroporation at 25 min cannot be explained by transient resealing (Figure 4, dashed line from 2.6 kV/cm), CA transport kinetics across the membrane could provide an answer.

When comparing transport kinetics of CA across membrane and transport kinetics of fluorescent dyes of similar size such as YO-PRO-1, it is important to consider the importance of size and charge of molecule in question.^40^ The transport of neutral CA molecules across the membrane is governed solely by chemical gradients, while the transport of positively charged YO-PRO-1 molecules across the membrane is governed by electrochemical gradient i.e. in addition to the concentration gradient transport is facilitated by the transmembrane voltage. These differences in driving forces of CA and YO-PRO-1 into the cell could also explain for plateau from CA detection experiments being shifted towards higher electric fields compared to plateau obtained from permeabilization experiments. We performed additional T_1_ relaxation measurements at 24 h after pulse delivery, i.e. at the same time when survival studies were performed. Results of CA detection after 24 h showed smaller decrease of T_1_ relaxation times in range of irreversible electroporation (at 2.6 kV/cm and at 3.0 kV/cm) compared to results at 25 min after pulse delivery. This smaller decrease of T_1_ relaxation time indicates that there was less CA present in suspensions that were exposed to higher electric fields 24 h after delivery of electric pulses. To further evaluate kinetics of CA transport across the membrane, average intracellular concentration of CA, at electric fields where plateau is reached (above 1.2 kV/cm), was calculated by combining relaxivity value of CA, T_1_ relaxation time of the control, T_1_ relaxation time of sample of interest and known cell volume fraction. We determined the average intracellular concentration of CA is approximately 1 μmol/L which is an order of magnitude lower compared to concentration of CA in electroporation medium (22 μmol/L). This can explain that exit of CA from cells is slower compared to its entry into the cell due to lower chemical gradient i.e. smaller difference in CA concentrations. Moreover, since transport of CA across the membrane is governed by chemical gradient only, transport occurs in both directions (i.e. extra- to intracellular during the initial phase immediately after electroporation and intra- to extracellular after CA washing and cell having membrane integrity compromised).

Electroporation outcome can reliably be assessed by evaluating temporary increase in cell membrane permeability using hydrophilic fluorescent dyes such as YO-PRO-1 and propidium iodide.^23,41^ However, this method can only be applied *in vivo* through histological analysis of treated tissue after animal euthanasia, making it unfavourable to use for investigations *in vivo*. Also, both YO-PRO-1 and propidium iodide bind to the nucleic acids once inside the cell, preventing them from exiting the cell even if the cell membrane is not resealed. This renders them ineffective in distinguishing between reversible and irreversible electroporation. Furthermore, difficult assessment of electric fields *in situ*^42^, is hindering clinical implementation of electroporation-based therapies and treatments despite great efforts and advancements in treatment planning.^43,44^ In contrast, the CA entrapment method of electroporation threshold detection employs similar concepts to fluorescent dye use for detecting changes in cell membrane permeability *in vitro* and can be imaged noninvasively using MRI scanner. Given that numerous factors affect cell membrane electroporation, including pulse characteristics and cell types, additional studies involving different cell models and pulse protocols are warranted to validate the universality of the CA entrapment method for electroporation detection. Nevertheless, the applicability of CA entrapment detection in clinical settings in future seems feasible, given the safety of CAs, as their surrounding chelate cage prevents interaction with biological structures.^45^ Nonetheless, further research on the safety of CAs in intracellular environment is needed.
